# Acute glycemic control in diabetics. How sweet is oprimal? Con: Just as sweet as in nondiabetic is better

**DOI:** 10.1186/s40560-018-0337-1

**Published:** 2018-11-06

**Authors:** Moritoki Egi

**Affiliations:** Department of Anesthesiology, Kobe University Hospital, 7 -5-1 Kusunoki-cho, Chuo-ku, Kobe City, 650-0017 Japan

**Keywords:** Diabetes, Chronic, Hyperglycemia, Liberal

## Abstract

This review is for Con side of “Pro-Con debate” on the optimal target of blood glucose levels in patients with chronic hyperglycemia (e.g. premorbid HbA1c level > 7%). Currently, international guideline recommended that blood glucose level ≤ 180 mg/dL in critically ill patients irrespective of presence or absence of premorbid diabetes. However, there are several studies to generate the hypothesis that liberal glycemic control (e.g., target blood glucose level 180–250 mg/dL) may be beneficial in critically ill patients with premorbid hyperglycemia. Although there is before-after study to report its safety and feasibility, it should be noted that this strategy may have a potential to increase the risk of infection, glycosuria, and polyneuropathy. Furthermore, there is randomized controlled study which showed the potential harm of liberal glycemic control in patients with premorbid hyperglycemia. Additionally, there are lots of uncertainty about the candidate and methodology of such a permissive hyperglycemia. With considering these facts, it might be better to keep target of blood glucose level in patients with diabetes the same as patients without diabetes (≤ 180 mg/dL), until randomized control study as like LUCID (the Liberal GlUcose Control in Critically Ill Patients with Pre-existing Type 2 Diabetes) trial will justify its risk and benefit.

## Background

This review is one of “Pro-Con” reviews to discuss the optimal target of blood glucose levels in patients with chronic hyperglycemia (e.g., premorbid HbA1c level > 7%). It is for the “Con” side standing for the statement that optimal target of acute glycemic control in patients with chronic hyperglycemia was same as in non-diabetic patients (≤ 180 mg/dL).

Although intensive insulin therapy (target blood glucose 80–110 mg/dL) had been reported to lower the mortality in a single-center randomized controlled trial [1], cumulative evidences show that such a glycemic management had significantly higher incidence of hypoglycemia and no further merit on the mortality and morbidity. According to the results of NICE-SUGAR trial [2] and subsequent meta-analysis [3], international guideline for management of sepsis recommended to maintain blood glucose level ≤ 180 mg/dL in acute illness [4, 5].

## How differently the target of blood glucose level is recommended in patients with and without diabetes

NICE-SUGAR trial had reported that intensive glucose control increased mortality among adults in the ICU. In other words, a blood glucose target of ≤ 180 mg/dL resulted in lower mortality than did a target of 81 to 108 mg/dL [2]. This effect was not significantly different between patients with and without diabetes (*p* = 0.60). Recently, one study reported estimation of optimal blood glucose level in critically ill patients using network meta-analysis [6]. However, this study could not analyze the optimal target in acute ill patients with premorbid diabetes due to the limit of evidence. Accordingly, current guideline recommends the same target of blood glucose level (≤ 180 mg/dL) irrespective of presence or absence of premorbid diabetes [5].

## The liberal glycemic control in critically ill patients with chronic hyperglycemia

There are studies shown that relationship between hyperglycemia and outcomes was altered by the presence of diabetes mellitus [7–12]. Furthermore, there are studies reported that premorbid hyperglycemia might interact the relationship between acute glycemic control and mortality [13–15]. Accordingly, these observational studies suggest that a liberal glycemic level (e.g., between 180 and 250 mg/dL) in critically ill patients with chronic hyperglycemia may be beneficial [16–19].

However, there are limited controlled studies to justify the benefit or harm of such a liberal glycemic control in particular cohort. Recent before-after study conducted in critically ill patients with diabetes shows that liberal glucose control was associated with decrease in insulin administration without any difference on clinical outcomes. This study also showed that the incidence of hypoglycemia was decreased in patients with chronic hyperglycemia [20]. However, there are several concerns on such a “permissive hyperglycemia” in critically ill patients with diabetes.

## There are concerns of liberal glycemic control in patients with diabetes

First concern on the “permissive hyperglycemia” in patients with diabetes is the risk of infection. Rayfield et al. had reported that there is significant association of mean glycemia and the risk of infection in diabetic patients [21]. There is a diminution in intracellular bactericidal activity of leukocytes and lower serum opsonic activity for bacteria in patients with poorly controlled diabetes. It should be noted that Centers for Disease Control and Prevention Guideline for the prevention of surgical site infection recommends to avoid the hyperglycemia as 200 mg/dL in patients with and without diabetes (category IA—strong recommendation; high to moderate—quality evidence) [22].

Second concern is the risk of glycosuria. Ruhnau et al. conducted prospective study to assess the renal threshold for glucose in patients with non-insulin-dependent diabetes [23]. At the level of 180 mg/dL of blood glucose, about half of the patients had partial glycosuria and the rest had no glycosuria. However, at the level of 250 mg/dL, approximately two thirds of the patients had persistent glycosuria. In critically ill patients, to maintain intravenous blood volume is relevant. Therefore, we might be able to prevent the glycosuria accompanied with permissive hyperglycemia.

Third concern is the risk of polyneuropathy. The polyneuropathy is common in patients with longer duration of diabetes and chronic hyperglycemia [24]. The analysis of pooled dataset of two randomized controlled trials shows that lowering blood glucose control had a non-significant trend to decrease the incidence of critical illness-induced polyneuropathy in patients with diabetes (43.9% vs 32.6%; odds ratio = 0.62, *p* = 0.25) [25]. These findings may suggest that hyperglycemia might be better to be avoided to prevent polyneuropathy in critically ill patients with diabetes.

In different words, these three concerns might suggest that conventional control may be beneficial to lower the risk of infection, to avoid the derangement due to the glycosuria, and to prevent the polyneuropathy in comparison with the liberal glycemic control in acute ill patients with chronic hyperglycemia.

## The randomized controlled trial to assess “permissive hyperglycemia” in acute ill patients with hyperglycemia

The DIGAMI study is a multicenter randomized controlled trial comparing between blood glucose level < 198 mg/dL and no use of insulin in post-myocardial infarction patients with HbA1c of around 8% [26]. The blood glucose level 24 h after randomization in no insulin group was 211 mg/dL in average, which is significantly higher than those of 173 mg/dL in the group of < 198 mg/dL. In the DIGAMI study, blood glucose control < 198 mg/dL significantly reduced 1-year mortality in comparison with those without using insulin. As DIGAMI study was conducted 25 years ago, their finding may not be generalized into current practice. Nonetheless, we should note that there is interventional study to show that the permissive hyperglycemia may increase the mortality in comparison with current usual glycemic control in patients with premorbid hyperglycemia.

## Conclusion

Liberal glycemic control is the concept of permissive acute hyperglycemia in critically ill patients with premorbid hyperglycemia. Although there are several studies to support this hypothesis and to report its safety and feasibility, we should note that this strategy may have a potential to increase the risk of infection, glycosuria, and polyneuropathy. Furthermore, there is randomized controlled study which showed the potential harm of liberal glycemic control in patients with premorbid hyperglycemia. Additionally, there are lots of uncertainty about the candidate and methodology of such a permissive hyperglycemia.

Considering above facts, it might be better to keep target of blood glucose level in patients with diabetes as same as in patients without diabetes (≤ 180 mg/dL), until randomized control study as like LUCID (the Liberal GlUcose Control in Critically Ill Patients with Pre-existing Type 2 Diabetes) trial justify its risk and benefit.
